# Differences in Electrical Delay Between the Mid-septum and Apex with the Right Ventricular Lead: Novel Implications for Pacemaker Sensing

**DOI:** 10.19102/icrm.2023.14034

**Published:** 2023-03-15

**Authors:** Fani Zagkli, Katerina Trigka, Rafail Koros, Panagiotis Chronopoulos, John Chiladakis

**Affiliations:** ^1^University Hospital of Patras, Department of Cardiology, Greece

**Keywords:** Apex, electrical delay, mid-septum, pacemaker, right ventricular lead

## Abstract

Ventricular sensing relies on the analysis of a local intracardiac electrogram in reference to the QRS on the surface electrocardiogram. If both signals do not coincide in time, there is a delay in sensing intrinsic ventricular activity. We evaluated possible differences in the electrical delay between the mid-septum and apex as determined by the right ventricular (RV) lead position using a pacing system analyzer (PSA) during conventional pacemaker implantation. Patients without significant heart disease and intrinsic atrioventricular conduction underwent their first Medtronic (Minneapolis, MN, USA) or Abbott (Chicago, IL, USA) dual-chamber pacemaker implantation with the RV lead first positioned at the apex and then subsequently at the mid-septum. Real-time ventricular sensing data were obtained through PSA to determine the electrical delay Q-VS value as the time difference between the QRS and the released RV-sensed event marker “VS.” Among 212 patients, 139 had narrow QRS and 73 had complete right bundle branch block (RBBB). Overall, both narrow QRS and RBBB patients exhibited shorter Q-VS lengths at the mid-septum compared to the apex (50.4 ± 24.2 ms and 66.7 ± 32.3 ms vs. 63.9 ± 27.6 ms and 71.7 ± 32.2 ms; *P* < .0001 and *P* < .001, respectively). The Q-VS in patients with Abbott devices was significantly shorter compared to that in patients with Medtronic devices at both the mid-septum and the apex in both patient groups (*P* < .0001). In conclusion, RV lead positioning at the mid-septum is associated with a shorter electrical delay compared to positioning at the apex in both narrow QRS and RBBB patients.

## Introduction

Sensing in pacemaker therapy means the ability of the device to recognize the heart’s intrinsic signal through the implanted lead by analyzing the local endocardial electrogram (EGM) with specific reference to the QRS on the surface electrocardiogram (ECG).^1^ Knowledge of the time course of the propagation of right ventricular (RV) electrical activation during intrinsic conduction is of great value for the understanding of ventricular sensing. With endocardial electrode catheters, early studies have demonstrated significant differences in the time of ventricular activation at the different RV anatomical locations, including early septal excitation starting at the left ventricular side and spreading to the right, activation of the RV apex before the RV outflow tract, and delayed RV activation in the presence of complete right bundle branch block (RBBB).^2–5^ Similar conclusions have been drawn more recently with 3-dimensional catheter-based mapping techniques.^6^ On this basis, one might speculate that differences in the timing of RV local activation at different locations correspond to different electrical delays in sensing through the RV lead. This proof may raise important issues related to the management of intrinsic ventricular activity or the occurrence of ventricular fusion/pseudofusion, a common pitfall in device follow-up, as not only pacemakers but also cardioverter-defibrillators and devices for cardiac resynchronization therapy (CRT) use the RV lead itself for sensing.^7,8^ Yet, basic knowledge regarding the relationship in time between QRS and acquired EGM from the RV lead is lacking in both narrow QRS and RBBB. One question of particular interest is the time at which RV lead sensing occurs at the mid-septum, which is nowadays considered the main alternative to the undesirable RV apical site for conventional pacemaker implantation.^9^

This study was performed to determine potential electrical delays in RV lead sensing at the mid-septum and the apex in patients with intrinsic narrow QRS or RBBB undergoing routine pacemaker implantation. As usual, RV sensing was intraoperatively tested by using a pacing system analyzer (PSA). The electrical delay (Q-VS) was calculated as the time between the reference QRS and the sensed EGM by the PSA (“VS”). Apical electrical delay Q-VS data were compared with those obtained by the same RV lead placed at the mid-septum in intraindividual comparisons.

## Methods

### Baseline characteristics

The study population consisted of 212 patients with normal left ventricular ejection fraction in the absence of significant structural heart disease who underwent their first conventional transvenous dual-chamber pacemaker implantation for standard indication after giving written informed consent. Eligible patients were hemodynamically stable and asymptomatic during the operation and had preserved intrinsic atrioventricular (AV) conduction of >40 bpm due to sinus bradycardia or second-degree AV block type II, with conducted narrow QRS (<120 ms), or wide QRS (≥120 ms) of RBBB configuration. Patients did not have a history of cardiovascular or systemic disease, severe pulmonary or kidney disease, or heart failure but had normal cardiac chamber size and left ventricular systolic function as assessed by M-mode and 2-dimensional echocardiograms. Patients exhibiting left bundle branch block, other intraventricular conduction disturbances with a pattern not conforming to RBBB, or third-degree AV block were excluded from this study. Patients were excluded if they had an atrial or ventricular arrhythmia, or if they needed either sympathomimetic drugs or atropine or temporary ventricular pacing during surgery. Patients undergoing pulse generator change or lead revision were also excluded. Based on the baseline ECG before the operation, the patients were separated into 2 groups according to narrow QRS or RBBB recognized by signs of delay in RV depolarization.^10^

### Procedure technique and electrical testing

**Figure 1:**
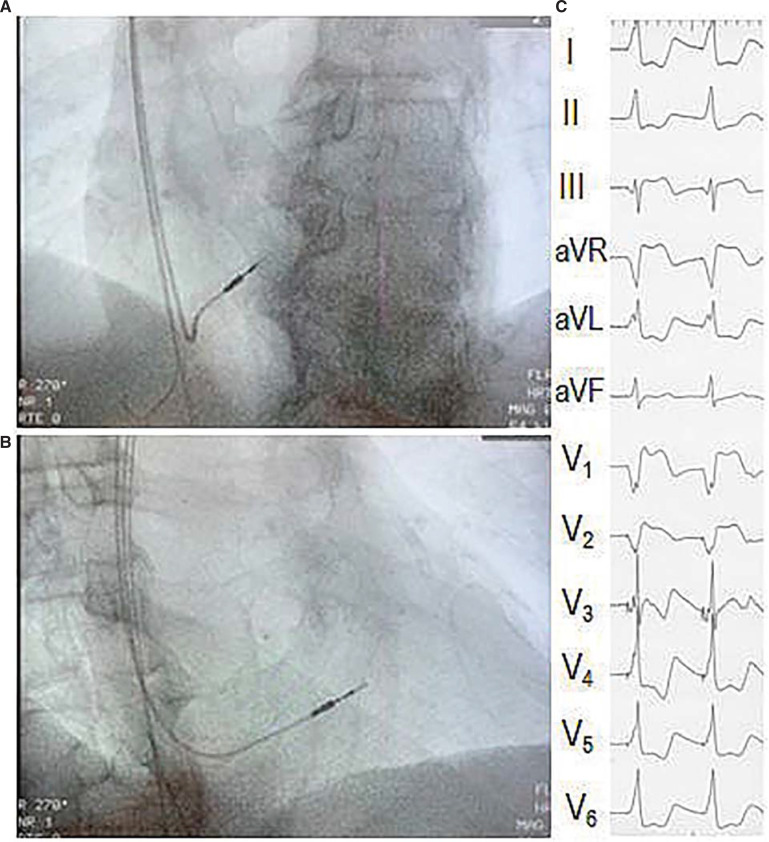
Fluoroscopic and electrocardiographic landmarks of right ventricular mid-septal lead placement. **A:** The lead tip is pointing in the left anterior oblique 30° view posteriorly toward the spinal column. **B:** In the anterior oblique 30° view, the lead tip is located at the center of the heart silhouette. **C:** Paced QRS morphology with early transition in the precordial leads.

Implantation of a Medtronic (Minneapolis, MN, USA) or Abbott (Chicago, IL, USA) pacemaker generator was performed under continuous ECG monitoring through an external surface 12-lead ECG polygraph (WorkMate Claris™; Abbott). Standard implant techniques were used with local anesthesia. Active-fixation leads were placed in the right atrium and the right ventricle. A commercially available bipolar steroid-eluting RV lead of the same brand (Medtronic™ model 4076 with tip electrode surface area of 4.2 mm^2^ or Abbott™ model 2088 with tip electrode surface area 6.9 mm^2^; both 58 cm in length with a tip-to-ring spacing of 10 mm) was used. The RV lead was initially positioned near the RV apex where the first measurements were taken. It was then advanced into the pulmonary artery and, after exchanging the stylet with another bent distal with posterior angulation, it was pulled back by fluoroscopy guidance to fall below the RV outflow tract (mid-septum), at the center of a radiographic 9-square grid. The mid-septal position was verified by combined fluoroscopy views and characteristic ECG of the early transition zone in the precordial paced QRS, as demonstrated in **Figure 1**.^11^

Programmers’ screens (Medtronic™ CareLink 2090 with 2290 analyzer or Abbott Merlin™ PCS) were configured to display simultaneous ECG lead II, markers, and RV EGM recordings on rhythm strips in real time. The standard programmer’s ECG cables and lead wires of Medtronic™ and Abbott™ manufacturers (5.4 m and 4 m in length, respectively) connected the programmers to skin electrodes for ECG recording. The use of a PSA (Medtronic™ model 2290 or Abbott™ model EX3100) of the same pacemaker brand was considered for traditional sensing measurements. PSA cable connections (Medtronic™ model 5833SL measuring 3.66 m in length or Abbott model 4051L measuring 3.7 m in length) were used to acquire the EGM in bipolar mode by using crocodile clips on the terminal pins of the RV lead.

**Figure 2:**
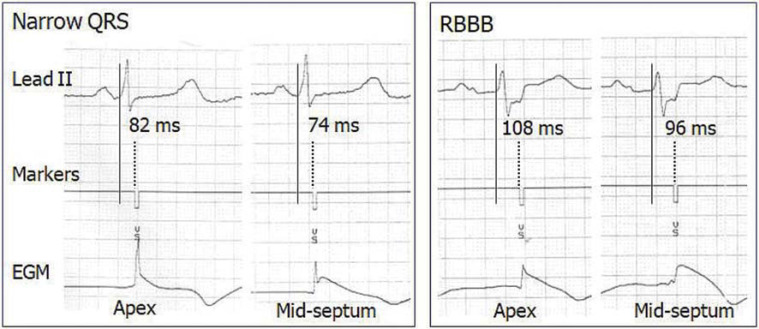
Representative cases of right ventricular lead electrical delay on the mid-septum and the apex with use of a Medtronic pacing system analyzer. Real-time electrograms in narrow QRS (left panel) and right bundle branch block (right panel) at a sweep speed of 50 mm/s. The calipers are aligned with the onset of the QRS complex in lead II (continuous vertical line) and the onset of the ventricular sensed event marker (dotted vertical line). Note the shorter right ventricular lead electrical delay between the QRS and the ventricular sensed event marker on the mid-septum compared to that on the apex in either narrow QRS or right bundle branch block. *Abbreviations:* EGM, intracardiac electrogram; RBBB, right bundle branch block.

**Figure 3:**
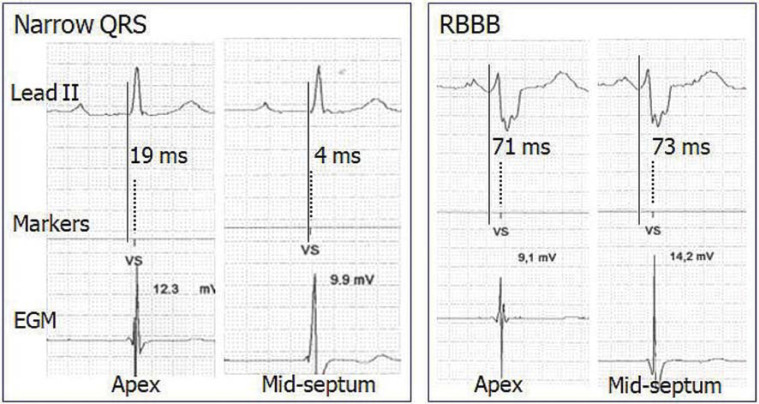
Representative cases of right ventricular lead electrical delay on the mid-septum and the apex with use of an Abbott pacing system analyzer in narrow QRS (left panel) and right bundle branch block (right panel). Note that the right ventricular lead electrical delay on the mid-septum compared to that on the apex is shorter in narrow QRS and slightly longer in right bundle branch block. *Abbreviations:* EGM, intracardiac electrogram; RBBB, right bundle branch block.

For baseline ECG analysis, a 12-lead intrinsic ECG was recorded before implantation at a paper speed of 50 mm/s with 10 mV/cm gain. The QRS duration was measured as the interval from the first onset of the QRS in any lead until the latest offset in any lead. During the PSA session, real-time rhythm strips were obtained through the programmer’s internal printer at a sweep speed of 50 mm/s comprising concurrent ECG lead II, marker telemetry, and EGM for Q-VS measurement. This was defined as the time delay difference between the beginning of QRS to the onset of the released ventricular sensed event marker “VS” at the corresponding apical versus mid-septal RV lead position if both recordings did not coincide **(Figures 2 and 3)**. Baseline ECG and rhythm strip data were analyzed manually over 3 cardiac cycles and then averaged using an electronic digitizer (Yansen Digital Caliper; Central Tools Inc., Cranston, RI, USA) by a single investigator, with an intraobserver variability of 0.89 ± 6.2 ms.

### Statistical analysis

Results of patient characteristics are presented as mean ± standard deviation values or as percentages. Appropriate parametric or non-parametric statistical tests (Student’s *t* test or Wilcoxon signed-rank test for paired data and Student’s *t* test or Mann–Whitney *U* test for unpaired data) were used for continuous variables and chi-squared tests were used for categorical variables, with a level of significance *P* < .05. Statistical analysis was performed using the SPSS statistical software package (version 24) (IBM Corporation, Armonk, NY, USA).

## Results

### Baseline characteristics and sensing measurements

Demographic and clinical parameters of the 212 study patients (mean age, 76 ± 9 years; range, 39–94 years) are summarized in **Table 1**. Of these, 139 (66%) had narrow QRS and 73 (34%) had RBBB isolated or associated with left anterior hemiblock. Both groups were comparable in terms of age, sex, and history of hypertension and diabetes. The indication for pacing was symptomatic sinus bradycardia in 72% of the patients and AV block in 28%. A similar proportion of narrow QRS and RBBB patients were taking angiotensin-converting enzyme inhibitors or calcium-channel blockers (42% and 47%, respectively; *P* = .4).

Patients with narrow QRS had a higher heart rate (*P* < .01) than RBBB patients. Overall, more Medtronic than Abbott pacemakers were allocated in the overall population (147/65, 69%) as well as separately in the narrow QRS (103/139, 74%) and the RBBB (44/73, 60%) patient groups.

Narrow QRS and RBBB patients had similar R-wave amplitudes and slew rates at both the mid-septum and the apex (10.3 ± 3.6 and 10.3 ± 3.6 mV vs. 10.2 ± 3.6 and 9.9 ± 4.2 mV and 2.8 ± 1.1 and 2.8 ± 1.2 V/s vs. 2.7 ± 0.9 and 2.6 ± 1.0 V/s, respectively; *P* = non-significant). There were no differences in R-wave amplitude or slew rate values obtained by the different PSAs when the study patients were analyzed as separate groups of narrow QRS patients versus RBBB patients (*P* > .5 for all comparisons).

### Electrical delay at the mid-septum versus the apex

Data on the Q-VS results are presented in **Tables 1 and 2**. In the overall population, the Q-VS at the mid-septum was shorter compared to those at the apex (66.7 ± 29.5 ms; range, 0–118 ms vs. 56 ± 28.3 ms; range, 0–122 ms; *P* < .0001). Similarly, the separate groups of narrow QRS and RBBB showed a shorter Q-VS at the mid-septum compared to the apex (50.4 ± 24.2 and 66.7 ± 32.3 ms vs. 63.9 ± 27.6 and 71.7 ± 32.2 ms; *P* < .0001 and *P* < .001, respectively). In addition, the presence of RBBB contributed to a greater Q-VS at both the mid-septum and the apex (*P* < .05 and *P* < .001, respectively). Of note, on 5 occasions in the narrow QRS group (4%) and 24 occasions in the RBBB group (33%), the Q-VS interval at the mid-septum was found to be either equal or prolonged compared to that at the apex. Overall, the average absolute Q-VS difference between the mid-septum and apex was 13.5 ms in narrow QRS patients and 5 ms in RBBB patients. In almost all cases, display of the “VS” marker by the RV lead was delayed in relation to QRS onset; only a few instances of nearly simultaneous records were seen.

**Table 2** shows the results of the electrical delay according to the PSA manufacturer. Overall, the Q-VS at the mid-septum was shorter compared to that at the apex in both patient groups with either Medtronic or Abbott devices (*P* < .05). Longer mid-septal than apical Q-VS periods were found in 2 of 103 Medtronic patients (2%) and 1 of 36 Abbott patients (3%) with narrow QRS and 15 of 44 Medtronic patients (34%) and 9 of 29 Abbott patients (31%) with RBBB, respectively. The average absolute difference in Q-VS between the mid-septum and apex with Medtronic or Abbott devices was 15.7 and 7.1 ms in normal QRS patients and 5.8 and 4.2 ms in RBBB patients, respectively. The Abbott device population’s Q-VS was significantly shorter compared to that of the Medtronic device population at both the mid-septum and the apex in both patient groups (*P* < .0001).

## Discussion

The specific aim of this study was to compare systematically the RV lead electrical delay at the mid-septum versus the apex using a PSA during conventional pacemaker implantation. To the best of our knowledge, this report verified for the first time that placement of the RV lead at the mid-septum is associated with a shorter electrical delay compared to placement at the apex in both narrow QRS and RBBB patients.

Timing differences in RV excitation have long been known about in both patients with normal QRS and RBBB.^2–5^ In prior studies, on average, the activation front appeared to arrive in normal QRS patients at the RV apex in 18 ms and at the RV outflow tract in 33–40 ms, whereas, in RBBB patients, it arrived at 51–54 ms and 78–84 ms, respectively. To date, little is known about the importance of the electrical delay at different RV lead locations of implanted devices. Previous studies have mainly focused on use of the electrical interventricular delay between the right and the left ventricular leads as an indicator of a better CRT response.^12,13^

While there is no doubt that the device’s sensing function is a daunting engineering undertaking, the clinician’s intraoperative task is easy, usually involving only the measurement of amplitude and the slew rate of the endocardial EGM signal by the PSA. In this regard, following implantation, the implanted pulse generator is expected to offer sensing data similar to those obtained intraoperatively. This study questioned the accuracy of the sensing performance in the time measured by the PSA, as determined by the specific RV lead position. If the released RV-sensed event marker “VS” does not coincide in time with the reference QRS complex on the ECG, timing information for accurate sensing local ventricular activity could have become lost. Nevertheless, a time lag for ventricular sensing is certainly real for a great variety of reasons. These involve possibly different raw materials in lead and device construction as well as different QRS recognition methods and band-pass filters for processing sensing EGM.

In line with the theoretical background of the spread of ventricular excitation,^2–5^ our results verified that placement of the RV lead at the mid-septum entailed a shorter electrical delay compared to that at the RV apical position in patients with structurally normal hearts and either narrow QRS or RBBB. Overall, we found that the absolute timing difference of the electrical delay between the mid-septum and apex averaged 13.5 ms in narrow QRS and 5 ms in RBBB patients, respectively. These differences might be considered small, particularly when using the Abbott PSA system, but they were statistically significant. Further, the substantial interpatient electrical delay variability measured by the PSA of either manufacturer suggests individualized assessment and tailored AV delay programming.

### Clinical implications in pacemaker therapy

The clinical consequences of RV lead electrical delay largely relate to the concept of preventing unnecessary ventricular pacing. With increased electrical delay, the sensed AV interval becomes more prolonged and the ventricular channel waits longer to alert for potential incoming ventricular activity. A longer sensed AV interval may be beneficial by allowing more intrinsic ventricular activity in patients who are not pacemaker-dependent, supporting the concept of ventricular pacing minimization.^14,15^ This should be particularly considered when autointrinsic conduction search algorithms are not available or activated or the clinician uses manual programming and fixed long AV intervals in patients with intact, low-degree, or intermittent AV conduction. On the other hand, the electrical delay may allow fusion/pseudofusion to occur, which in turn may confound special pacemaker functions, such as wasting battery due to repetitive pacing threshold tests or falsely counting intrinsic beats as ventricular paced events.^7,16^ Thus, if a sensing delay is present, it needs to be carefully tailored to the individual AV delay. In point of fact, programming a longer AV delay, at least to the value of RV lead electrical delay, may encourage intrinsic ventricular activity. Conversely, a shorter sensed AV interval may be allowed in CRT recipients to increase true biventricular pacing.

In RBBB, the well-known phenomenon of non-sensing by the RV lead due to the presence of ipsilateral bundle branch block or ectopic depolarization arising from the contralateral ventricle is based on the concept of delayed RV activation because the activation front spreads from the left ventricle toward the RV lead recording site. A longer programmed sensed AV interval than the baseline apparent P–R interval is generally recommended in dual-chamber pacing to avoid loss of sensing or inappropriate ventricular pacing.^17^ This might not be needed to be prolonged far beyond the apparent P–R interval if the RV lead is positioned at the mid-septum.

### Limitations

In the present study, selective RV lead placement at the apex and mid-septum was chosen, and the effect of RV lead placement on the electrical delay at other alternative RV sites was not assessed. Also, the sensing behavior of the pacemaker generator sensing circuit instead of the PSA may be different. In addition, the extrapolation of our results from our select population to patients with structural heart disease is precarious. Finally, we did not analyze patients with isolated RBBB and those with concomitant left anterior hemiblock separately. However, given the very modest RV sensing delay differences between the mid-septum and apex of ≤5 ms, we postulate that a separate assessment of these conduction disturbances would not lead to notable sensing delay differences.

## Conclusion

RV lead placement at the mid-septum enables shorter electrical delays than those at the apex in both narrow QRS and RBBB. This result strengthens the choice of an RV lead mid-septal position as regards the accuracy of the device’s sensing function in time. One must be aware that the individual sensed AV delay may vary among device manufacturers and ECG features. In dual-chamber pacing, by remembering about the RV lead electrical delay, the extension of the sensed AV delay to the value of electrical delay may contribute to the avoidance of unnecessary ventricular pacing.

## Figures and Tables

**Table 1:** Patient and Procedural Characteristics

Variable	Total (n = 212)	Narrow QRS (n = 139)	RBBB (n = 73)	*P* Value
Age (years)	76 ± 9	75 ± 9	77 ± 8	NS
Male sex, n (%)	126 (59)	81 (58)	45 (62)	NS
Body mass index (kg/m^2^)	27.6 ± 4.1	27.3 ± 4.1	28.2 ± 4.1	NS
Hypertension	98 (46)	61 (44)	37 (51)	NS
Diabetes	62 (29)	38 (27)	24 (33)	NS
Heart rhythm, n (%)				
Sinus bradycardia	152 (72)	117 (84)	35 (48)	<.0001
AV conduction disease	60 (28)	22 (16)	38 (52)	<.0001
ECG at baseline				
HR (beats/min)	59.8 ± 13.1	61.7 ± 12.8	56.2 ± 13.2	<.01
P–R interval (ms)	205 ± 43	205 ± 45	206 ± 39	NS
QRS duration (ms)	114 ± 29	94 ± 11	151 ± 13	<.0001
Electrical delay, Q-VS (ms)				
Mid-septum	56.0 ± 28.4	50.4 ± 24.2	66.7 ± 32.3	<.001
Apex	66.7 ± 29.5	63.9 ± 27.6	71.7 ± 32.2	<.05

*Abbreviations:* AV, atrioventricular; ECG, electrocardiogram; HR, heart rate; NS, not significant; RBBB, right bundle branch block. Values are presented as mean ± standard deviation or as n (%).

**Table 2:** Electrical Delay of Apical and Mid-septal Right Ventricular Lead Positions

Q-VS interval	Apex	Mid-septum	****P**** Value
Narrow QRS			
Medtronic (n = 103)	77.8 ± 16.1 (18–112)	62.1 ± 15.3 (15–100)	<.0001
Abbott (n = 36)	24.2 ± 10.3 (0–49)	17.1 ± 9.8 (0–42)	<.0001
RBBB			
Medtronic (n = 44)	94.7 ± 15.3 (58–118)	88.9 ± 17.5 (37–122)	<.01
Abbott (n = 29)	36.9 ± 15.9 (2–72)	32.7 ± 16.2 (2–65)	<.05

*Abbreviation:* RBBB, right bundle branch block. Values are presented as mean ± standard deviation (range).
